# Genomic and resistome analysis of *Alcaligenes faecalis* strain PGB1 by Nanopore MinION and Illumina Technologies

**DOI:** 10.1186/s12864-022-08507-7

**Published:** 2022-04-20

**Authors:** Jidong Lang, Yanju Li, Wenjuan Yang, Ruyi Dong, Yuebin Liang, Jia Liu, Lanyou Chen, Weiwei Wang, Binbin Ji, Geng Tian, Nanying Che, Bo Meng

**Affiliations:** 1 Geneis (Beijing) Co., Ltd, Beijing, 100102 China; 2 School of Life Science, Beijing Institute of Technology, Beijing, 100081 China; 3 Department of Pathology, Beijing Key Laboratory for Drug Resistant Tuberculosis Research, Beijing Chest Hospital, Capital Medical University, Beijing Tuberculosis and Thoracic Tumor Research Institute, Beijing, 101149 China; 4 Qingdao Geneis Institute of Big Data Mining and Precision Medicine, Qingdao, People’s Republic of China

**Keywords:** *Alcaligenes faecalis*, Antibiotic-resistant, Genome assembly, Next-generation sequencing, ONT sequencing

## Abstract

**Background:**

Drug-resistant bacteria are important carriers of antibiotic-resistant genes (ARGs). This fact is crucial for the development of precise clinical drug treatment strategies. Long-read sequencing platforms such as the Oxford Nanopore sequencer can improve genome assembly efficiency particularly when they are combined with short-read sequencing data.

**Results:**

*Alcaligenes faecalis* PGB1 was isolated and identified with resistance to penicillin and three other antibiotics. After being sequenced by Nanopore MinION and Illumina sequencer, its entire genome was hybrid-assembled. One chromosome and one plasmid was assembled and annotated with 4,433 genes (including 91 RNA genes). Function annotation and comparison between strains were performed. A phylogenetic analysis revealed that it was closest to *A. faecalis ZD02*. Resistome related sequences was explored, including ARGs, Insert sequence, phage. Two plasmid aminoglycoside genes were determined to be acquired ARGs. The main ARG category was antibiotic efflux resistance and *β-lactamase* (EC 3.5.2.6) of PGB1 was assigned to *Class A, Subclass A1b, and Cluster LSBL3*.

**Conclusions:**

The present study identified the newly isolated bacterium *A. faecalis PGB1* and systematically annotated its genome sequence and ARGs.

**Supplementary Information:**

The online version contains supplementary material available at 10.1186/s12864-022-08507-7.

## Background

*Alcaligenes faecalis (A. faecalis)* is a Gram-negative, rod-shaped, motile, and obligate aerobe. As an oxidase-, catalase-, and citrate-positive bacteria, it is widely distributed in the soil, water, and elsewhere in the environment. Previous studies on *A. faecalis* focused mainly on its pollutant biodegradation/bioremediation capacity [1–3], induction of clinical infection [4, 5], genome assembly [6–8], bioremediation of phenols [9], polynuclear aromatic hydrocarbons [10], drug residues [11], and pesticides [12, 13]. Moreover, *A. faecalis* strains are widely used in agricultural and industrial processes [14]. Bharali P. et al. reported that an *A. faecalis* strain isolated from crude oil-contaminated soil produced biosurfactants and is a potential candidate for industrial-scale glycolipid biosurfactant fabrication. These materials are useful in various biotechnological and industrial processes especially in the petroleum industry [1]. A study by *Obata *et al*.* determined that *Alcaligenes* spp*.* can alter their morphology from rod- to coccoid for effective transfer into Peyer’s patches (PPs) in the soil [15]. *A. faecalis* converted highly toxic arsenite into relatively safer arsenate. It also tolerated heavy metals [7, 16]. Moreover, *A. faecalis* oxidizes indole [17], removes ammoniacal nitrogen [18], and biodegrades ochratoxin A and nicotinic acid [19, 20]. Hence, this organism has potential industrial benefits and is important to major spheres of research.

Opportunistic *A. faecalis* infections occured as this species is highly resistant to commonly used antibiotics [5, 21–24]. *A. faecalis* may be resistant to certain antibiotics but can also acquire antibiotic resistance via chromosomal gene mutations and horizontal gene transfer [25]. Several recent studies identified genes responsible for intrinsic resistance to *β-lactam*, *fluoroquinolone*, and *aminoglycoside antibiotics *[26–30]. *β-lactamases* such as *VIM-6*, *PER-1*, and *TEM-21* are key antibiotic resistance enzymes that have been detected in clinical *A. faecalis* isolates [31–34]. Therefore, analysis of the entire *A. faecalis* genome sequence might potentially disclose other antibiotic resistance genes and characterize the enzymes they encode.

To date, 47 *A. faecalis* strains have been sequenced and assembled. However, only eight strains have complete genome sequences and three have chromosome sequences. Though hundreds of discrete contigs were obtained, many of them were fragmented and incomplete because of sequence depth and the nature of short-read technologies [35]. A few recent studies demonstrated that third-generation sequencers such as those from PacBio and Oxford Nanopore Technologies (ONT) [36, 37] can facilitate assembly by generating sequencing reads ≤ 1 Mb long [38]. Longer reads enable the accurate interpretation of full bacterial genome structures including positional and structural delineations of bacterial antibiotic resistance islands [39]. The *E. coli* K-2 MG1655 chromosome was assembled de novo using a single 4.6-Mb contig obtained from ONT data. It had 99.5% nucleotide identity with its reference genome NC_000913.3 [38]. In another study, nine bacterial genomes were assembled including three *Aeromonas* strains, three *Flavobacterium* strains*,* and three *Pseudonocardia* bacteria. To this end, ONT reads were used and the assembly was polished with Illumina reads. A wide range of GC content was revealed and accurate annotation of difficult sequencing areas such as insertion sequences was achieved [39]. Wick et al. used the ONT platform to sequence 12 *Klebsiella pneumoniae* isolates on a single flow cell [35]. Thus far, however, there have been no reports of *A. faecalis* genome assembly on the ONT platform.

Here, we isolated a new *A. faecalis* strain, sequenced it on an ONT MinION sequencer, and constructed a paired-end library via an Illumina NextSeq sequencer. A hybrid assembly was made with published software. The assembled genome was annotated and the ARGs were discovered. Phylogenetic and genome comparison analyses were also conducted.

## Results

### Bacterial isolation and β-lactam antibiotic degradation

**Fig. 1:**
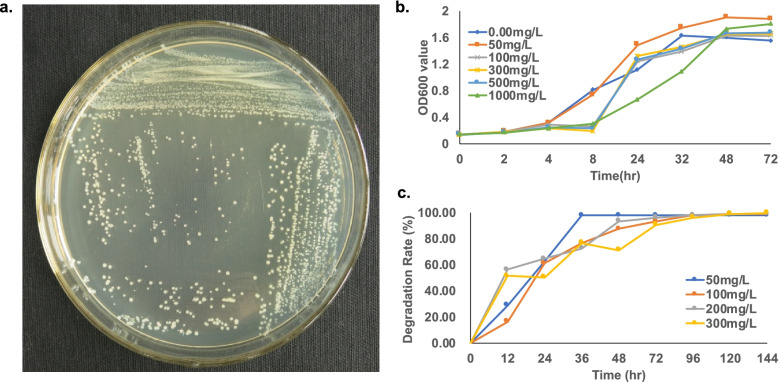
Alcaligenes faecalis Strain PGB1 growth condition images. **a.** colony morphology of *A. faecalis*, **b.** growth curve of *A. faecalis* under different penicillin concentrations, **c.** penicillin G degradation rates curve of *A. faecalis*

Here, we successfully isolated a penicillin-degrading bacteria (Fig. 1A) based on its clone morphology and 16S rDNA Sanger sequencing data (Additional file 2 Figures S1). It was identified as *A. faecalis* and named as strain PGB1. It was cultured using various penicillin G (PG) concentrations (0–1,000 mg/L) as the sole carbon source and its growth curve was plotted according to the OD_600_ measured at each PG concentration. The growth curve (Fig. 1B) was partitioned into three zones: low concentration (50 mg/L PG), medium concentration (100 mg/L, 300 mg/L, and 500 mg/L PG), and high concentration (1,000 mg/L PG). At low PG concentration, bacterial growth started to increase after 4 h. At all higher PG concentrations, bacterial growth started to increase after 8 h but at different acceleration rates. Although PGB1 grew under all tested conditions, very high PG concentrations reduced its replication rates and delayed the onset of increase in bacterial growth by ~ 4 h. By 144 h, the PG degradation rates at 50 mg/L, 100 mg/L, 200 mg/L, and 300 mg/L were 97.99%, 98.98%, 99.45%, and 99.65%, respectively (Fig. 1C).

PGB1 was also cultured in the presence of the *β-lactam* antibiotics cefalexin, cefradine, and cefuroxime sodium. After 96 h, the maximum degradation rates at 50 mg/L concentration were 57.18%, 70.86%, and 57.74% for cefalexin, cefradine, and cefuroxime sodium, respectively (Additional file 2 Figures S2).

### Sequence assembling comparison of the two sequencing technologies

Whole-genome sequencing of PGB1 was performed on the Illumina and ONT platforms. NextSeq Illumina 150-bp paired-end sequencing generated 17,201,542 reads (2.50 Gbp), with a 57% GC content. Low-quality clean reads were filtered and 17,198,815 high-quality reads (500 × coverage) were used for the assembly with SoapDenovo (k-mer length = 43) (Additional file 1 Tables S1). The mean read length was 128 bp and ~ 91.70% of the sequencing reads were > Q20 (Table 1).

**Table 1:** Short-read sequencing (Illumina) and long-read sequencing (ONT) data

Platform	Average Q score	Q20 Ratio	Average Length (bp)	Min Length (bp)	Max Length (bp)	Assemble Contig1 Length (bp)	Assemble Contig2 Length (bp)	K43 N50 Length (bp)	K43 Maximum Scaffold Length (bp)
Nanopore	18.9	–	4,848.50	106	136,627	4,239,915	174,141	–	–
Illumina	**-**	91.70%	128	50	131	–	–	18,013	69,386

**Fig. 2:**
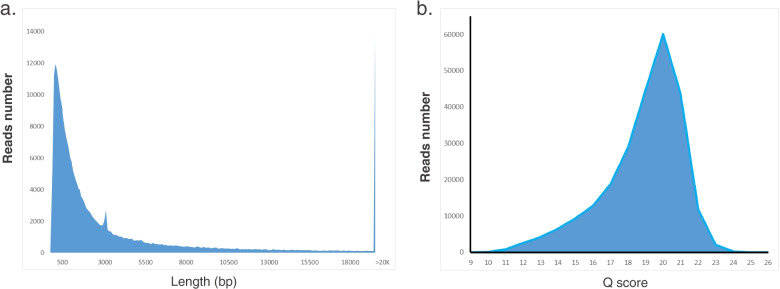
ONT sequencing read quality. **a** Read length distribution density plot. **b** Quality score distribution (Q-score ≥ 7)

ONT sequencing generated 246,674 reads (mean length = 4,848 bp) from 857 active pores (Table 1) with 56.83% GC content. The N50 read length for the dataset was 8,557 bp and its mean quality score was 18.90. Hence, ~ 271 × theoretical coverage was achieved (Table 1). Read lengths were in the range of 106–136,627 bp and the quality score was in the range of 9–25. A read length and quality score distribution map was shown in Fig. 2. Other sequence details are summarized in Table 1.

The Illumina sequencing reads were assembled into 506 scaffolds with N50 length = 18,013 bp and maximum scaffold length = 69,386 bp (Table 1). The ONT sequencing reads were assembled into two long scaffolds of which the longer was 4,239,915 bp. The shorter was 174,141 bp in length and shared 56% similarity with *A. faecalis* plasmid GZAF1 (pGZAF1). Thus, the ONT platform had significantly better assembly performance than the Illumina platform.

### *A. faecalis* PGB1 genome error corrections

CheckM was used to evaluate the Nanopore genome assembly and disclosed 81.66% completeness and 0.83% contamination. To correct base errors, fix misassemblies, and fill assembly gaps, we polished the ONT genome sequences in Pilon using Illumina PE reads (Table 2) [40]. The Illumina data confirmed > 90% of the ONT scaffold sequences and corrected several sequencing errors. For the longer scaffold (4,262,877 bp), polishing corrected 317 single-nucleotide polymorphisms (SNPs) and 1,022 small insertions containing 1,274 bases and 15,269 small deletions. The correction rate was 0.50%. The 175,292-bp scaffold had 17 SNPs, 59 small insertions, and 750 small deletions. Polishing provided a 0.47% correction rate. After polishing, the 4,262,877-bp ONT scaffold decreased by 22,962 bp to 4,239,915 bp and the 175,292-bp ONT scaffold decreased by 1,151 bp to 174,141 bp (Table 2).

**Table 2:** Draft genome obtained from ONT sequencing, Pilon polishing, and CheckM quality evaluation

Tool	Parameters	Chromosome	Plasmid-like DNA
FLYE	Length, bp	4,262,877	175,292
CheckM	CheckM genome size, bp	4,262,877	
	CheckM completeness	0.8166	
	CheckM contamination	0.0083	
	Strain heterogeneity	0	
Pilon	Length, bp	4,239,915	174,141
	Total reads	16,188,437	655,781
	Coverage	473	463
	MinDepth	47	46
	Confirmed bases	0.9915	0.9934
	Corrected SNPs	317	17
	Corrected ambiguous bases	1	0
	Corrected small insertions/total bases	1,022/1,274	59/86
	Small deletions	15,269	750
Polished CheckM	CheckM genome size, bp	4,239,915	
	CheckM completeness	0.9602	
	CheckM contamination	0.0029	
	Strain heterogeneity	0	

The CheckM results (Table 2) indicated that polishing significantly improved genome assembly quality. After polishing, genome completeness reached 96.02%. By comparison, it was only 81.66% before polishing and based on 419 gene markers (*C_Betaproteobacteia UID3959* marker lineage). Furthermore, polishing lowered the contamination rate from 0.83% to 0.29%. The strain heterogeneity was zero for both datasets.

### Genome annotation

**Fig. 3:**
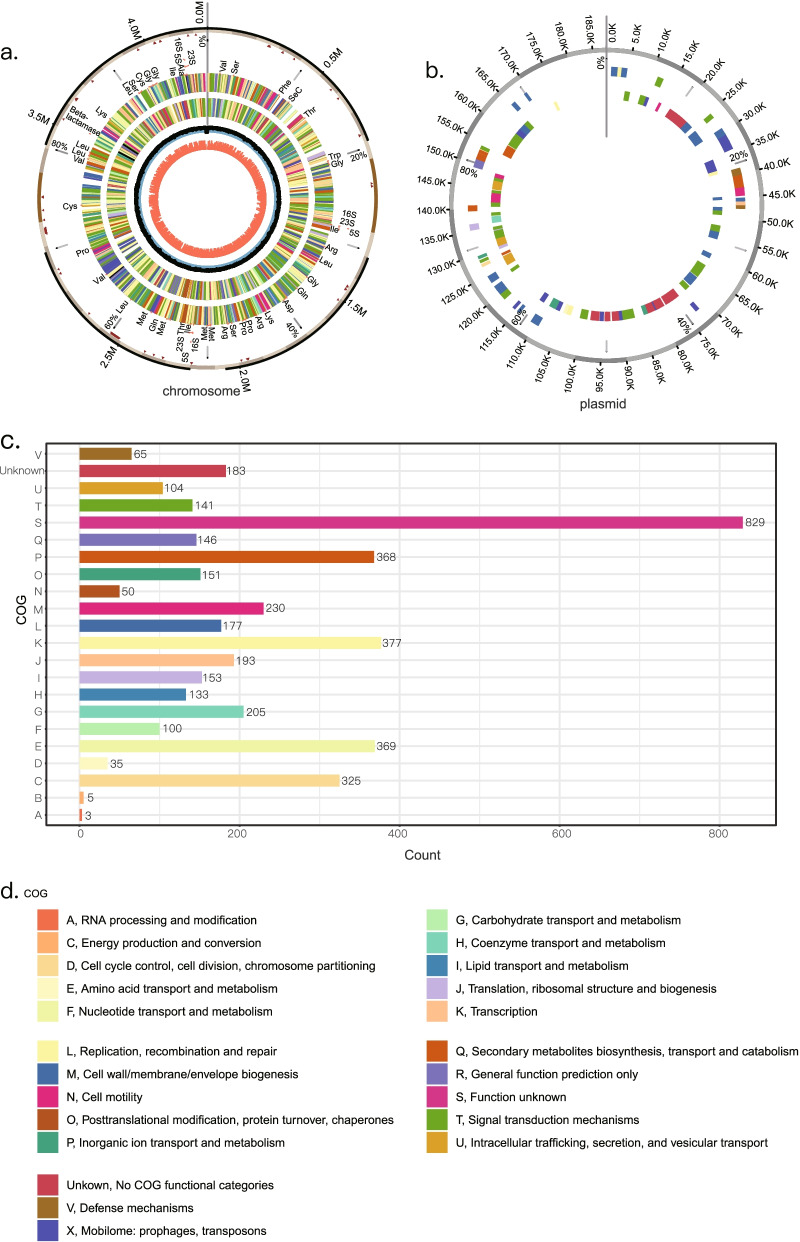
Graphical circular map representing *A. faecalis* PGB1 genome. **a**) *A. faecalis* PGB1 chromosome map. From outside to center: scale marks (resistance genes), RNA genes (tRNAs and rRNAs), genes on forward strand (color based on COG category), genes on reverse strand (color based on COG category), GC content, and GC skew (G-C) / (G + C). **b**) *A. faecalis* PGB1 plasmid-like DNA map. Outside circle shows scale (kb). Second and third rings show genes on forward and reverse strands. **c**) Gene numbers associated with general COG functional category predictions. **d**) COG figure legend

The circular genome map for *A. faecalis* PGB1 constructed by Circos displayed a 4,239,915-bp circular chromosome (Fig. 3a) and 174,141-bp plasmid DNA (Fig. 3b) with GC = 56.83% and 50.94%, respectively. The draft genome was predicted with Prodigal and annotated with eggNOG v. 5.0 (Additional file 1 Tables S2). The genome ncRNA was annotated by RNAmmer and Infernal. A total of 4,433 genes were predicted including 4,342 protein-coding (CDS), 9 rRNA, 58 tRNA, and 24 ncRNA genes (Tables 3 and Additional file 1 Tables S11–S13). The annotated genes were classified into four main functional categories including 21 subclasses (Fig. 3, Additional file 1 Tables S2) using the clusters of orthologous groups (COG) database. Each subclass had at ≥ 2 predicted genes. The largest subclass (S) had 831 genes (19.23%) of unknown function. The other top subclasses included K (*transcription*; 374 genes; 8.67%), P (*inorganic ion transport and metabolism*), E (*amino acid transportation*; 352 genes; 8.16%), C (*energy production and conversion*; 318 genes; 7.37%), and M (*cell wall/membrane/envelope biogenesis*; 230 genes; 5.33%). The top three COG subclasses with the fewest genes included RNA processing and modification (three genes: 0.07%), chromatin structure and dynamics (three genes; 0.07%), and cell cycle control, cell division, and chromosome partitioning (33 genes; 0.77%).

**Table 3:** Key features of *A. faecalis PGB1* genome

Features	Chromosome	Plasmid-like DNA
Total size, bp	4,239,915	174,141
Overall GC content	56.83%	50.94%
Total predicted genes	4,303	130
CDS	4,213	129
RNA genes	90	1
rRNA genes	9	0
5S rRNAs	3	0
16S rRNAs	3	0
23S rRNAs	3	0
tRNA genes	58	0
ncRNAs	23	1

We also examined gene distribution in the chromosomal and plasmid DNA forward and reverse strands (Fig. 3; Additional file 1 Tables S15). For the chromosome, there were ~ 2,023 and ~ 2,190 genes in the forward and reverse strands, respectively. The gene numbers and categories were more or less evenly distributed on both strands. For the plasmid-like DNA, the reverse strand had more annotated genes than the forward strand. For the plasmid DNA, there were 129 genes. Of these, 58.66% were as associated with replication, recombination, and repair (18.60%). Moreover, 25.58% were associated with intracellular trafficking, secretion, and vesicular transport, 3.10% were associated with post-translational modifications, protein turnover, and chaperones, 6.20% were associated with inorganic ion transport and metabolism, 5.40% were associated with signal transduction mechanisms, and 4.65% were associated with cell wall/membrane/envelope biogenesis. Another 10.07% of the plasmid-like DNA had unidentified functions.

Sixty-five genes were associated with defense mechanisms (Fig. 3). Most of these were related to multiple xenobiotic resistance. Hence, the bacteria could tolerate or even thrive in the presence of antibiotics and toxic compounds such as aminoglycoside hydroxyureas, acriflavine, and bacitracin. Most of the genes were located on the chromosome rather than the plasmid (Additional file 1 Tables S2). Hence, they were vital bacterial traits that required conservation and the risk of gene loss or transfer associated with plasmids could be avoided. The presence of genes associated with bioremediation capacity genomically explains the observed micropollutant tolerance in this versatile bacterium. Subsequent analyses were conducted by using the COG, gene ontology (GO), KEGG orthology (KO), and BiGG databases (Additional file 1 Tables S2).

### Phylogenetic tree analysis and genome structure and function comparison

The *A. faecalis* PGB1 was compared against 23 strains including 11 other *A. faecalis* whose complete genomes had already been published, two *E. coli*, and 10 others based on core genome analysis. Strain information is summarized in Additional file 1 Tables S3 and their NCBI GenBank numbers are listed. Thirty-three genes common to all strains were used in the analysis and are listed in Additional file 1 Tables S4. A 70% identity was used in the analysis.

**Fig. 4:**
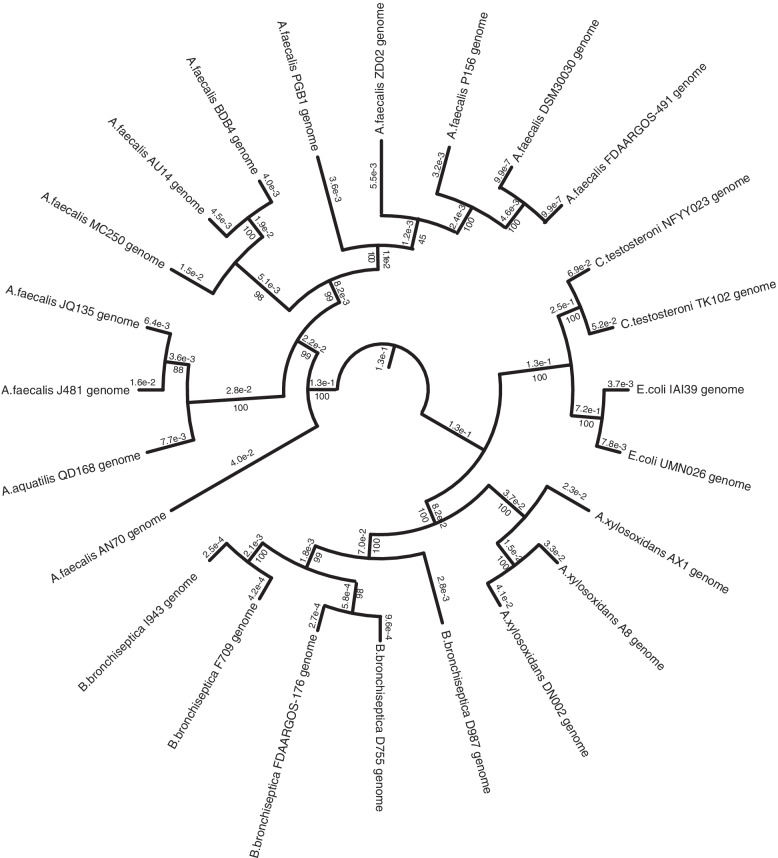
Phylogenetic tree of 24 genome sequences. Reference genome sequences were obtained from Genbank

The core phylogenetic tree (Fig. 4) showed that all 24 strains were classified into either the *A. faecalis* or the non*-A. faecalis* group. Strains of the same species clustered together into a group. Hence, the calculations were accurate. The ten *Alcaligenes* strains were divided into four clusters of which *A. faecalis* AN70 was separate from the others*.* Five *A. faecalis* (*P156, ZD02, PGB1, DSM30030, and FDAARGOS_491)* were clustered together and PGB1 was closest to *ZD02.* The *A. faecalis* strains *BDB4, AU14,* and *MC250* and the *A. faecalis* strains *JQ135, J481,* and *QD168* clustered individually into separate groups. The other four species were closely related to *E. coli, Comamonas testosteroni, Achromobacter xylosoxidans,* and *Bordetella bronchiseptica*. The calculated branch lengths shown in Fig. 4 indicate the exact evolutionary distances of the 24 bacterial strains.

To distinguish the *A. faecalis* strains, a genome annotation was performed with eggNOG on the other ten strains with complete genome or chromosome sequences. Detailed genome annotations and the number of genes per COG category are displayed in Additional file 1 Tables S5. All *A. faecalis* strains had similar genome sizes in the range of 3.87–4.25 Mbps. ZD02 was the largest strain followed by PGB1. The GC content of PGB1 was the highest. Based on the predicted genes and proteins, though ZD02 had the largest genome, PGB1 was annotated with the greatest number of genes and proteins. For unknown reasons, PGB1 and AU14 were missing 183 and 166 genes, respectively.

The phylogenetic tree disclosed that ZD02 was the closest strain to PGB1. Thus, we conducted synteny analyses on their genomes. Their sequence similarity was 95.77% but sequence inversions were detected in the PGB1 genome. Details of the sequence blast are summarized in Additional file 1 Tables S6.

### Antibiotic resistance traits of the *A. faecalis* PGB1 genome

To determine the antibiotic resistome characteristics of PGB1, we conducted a deep analysis of the antibiotic resistance genes. The PGB1 genome sequence was BLASTed using the AMR database. The predicted antibiotic resistance genes are shown in Table 4. Four genes were predicted including *aph(3')* and *bla* in the chromosome DNA and *aph(3’)-Ib*, *aph(6)-Id* in the plasmid. We also identified antibiotic resistance genes using the CARD database. We localized *adeF* in the resistance-nodulation-cell division (RND) antibiotic efflux pump family under tight cutoff conditions. This gene conferred fluoroquinolone and tetracycline resistance. We predicted 473 antibiotic resistance genes predicted under loose cutoff conditions. *Aph(3'')-Ib* was identified in the plasmid under tight cutoff conditions. It conferred resistance to aminoglycoside antibiotics. Seventeen genes were also found under loose cutoff conditions. In-depth exploration identified 299 genes in the antibiotic efflux mechanism group comprising > 50% of all genes predicted. The antibiotic target alteration and antibiotic inactivation groups contained 77 and 63 genes, respectively. Ten plasmid genes identified under loose cutoff conditions belonged to the antibiotic efflux group and six plasmid genes identified under loose cutoff conditions belonged to the antibiotic target alteration group (Additional file 1 Tables S7). Phage was predicted with PHASER. Two chromosome regions were predicted with an intact phage sequence. One region 15.6 kb in length had 22 CD matched with 16 phage types. The other region 37.2 kb long had 42 CDS matched with 32 phages. The ARGs *gadW* and *tetA* were predicted under loose cutoff conditions and detected within the 15.6-kb phage region. Five ARGs (*macB*, *AbaF*, *maxT*, *arlR*, and *basS*) were within the 37.2-kb phage region. An incomplete phage region 7.5 kb in length was identified in the plasmid. Detailed information was listed in Additional file 1 Tables S8. Acquired antibiotic resistance gene prediction was performed by Resfinder. The results (Additional file 1 Tables S9) revealed no acquired genes in the chromosome sequence but two acquired genes in the plasmid. The latter were aph(3'')-Ib in the 45,875–46,677 region and *aph(6)-Id* in the 46,677–47,511 region. Both of these were aminoglycoside resistance genes. We also performed insert sequence (IS) analyses on the PGB1 and ZD02 chromosomes and plasmid sequences (Additional file 1 Tables S10). Nineteen IS were detected in the PGB1 chromosome sequence. Of these, four (IS*Pa38*, *TnShfr1*, IS*Sod9*, and IS*Shes11*) belonged to the TN3 family and were identified in all four DNA sequences. IS*Azs17* was found in PGB1, ZD02, and ZD02-plasmid while IS*Pha2* and IS*Vsp2* were found in PGB1 and ZD02. The remaining 12 IS were localized only on the PGB1 chromosome. Each of the other three sequences was an unique IS. The 19 IS belonged to five families. Eleven of the 12 unique IS sequences in PGB1 belonged to the IS*3* family. Five *Tn3* family IS were detected in the PGB1 chromosome sequence and three of these were identified in all four sequences.

**Table 4:** Antibiotic resistance genes predicted by AMR database

Target identifier	Gene symbol	Protein name	Method	Target length	Reference protein length	% Coverage of reference protein	% Identity to reference protein	Alignment length	Accession of closest protein	Name of closest protein	HMM id	HMM description
PGB1_chr_1397	aph(3')	APH(3') family aminoglycoside O-phosphotransferase	HMM	257	NA	NA	NA	NA	NA	NA	NF033068.1	APH(3') family aminoglycoside O-phosphotransferase
PGB1_chr_3891	bla	class A beta-lactamase	HMM	292	NA	NA	NA	NA	NA	NA	NF033103.1	class A beta-lactamase
PGB1-pla_47	aph(3'')-Ib	aminoglycoside O-phosphotransferase APH(3'')-Ib	PARTIAL	140	267	52.06	100	139	WP_001082319.1	aminoglycoside O-phosphotransferase APH(3'')-Ib	NF032895.1	aminoglycoside O-phosphotransferase APH(3'')-Ib
PGB1-pla_48	aph(6)-Id	aminoglycoside O-phosphotransferase APH(6)-Id	PARTIAL	248	278	88.85	99.6	247	WP_000480968.1	aminoglycoside O-phosphotransferase APH(6)-Id	NF012171.0	APH(6)-I family aminoglycoside O-phosphotransferase

**Fig. 5:**
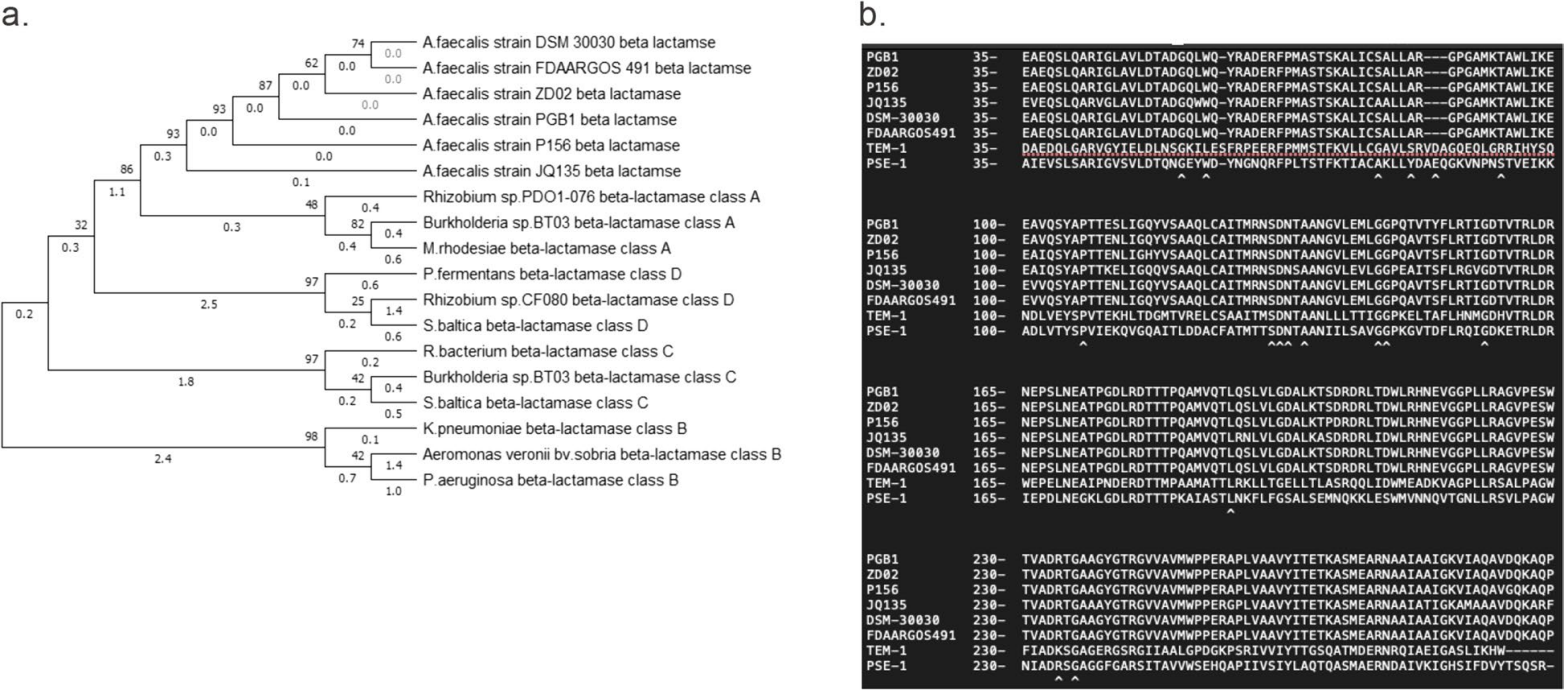
β-lactamase sequence alignments. **a**) Phylogenetic tree displaying relationships between β-lactamase (EC 3.5.2.6) encoded by *A. faecalis* PGB1 and other β-lactamases. Amino acid sequences are displayed above. **b**) Comparison of β-lactamase (EC 3.5.2.6) amino acid sequences encoded by *A. faecalis* with those conserved in class A, subclass A1b β-lactamases

Earlier studies showed that PGB1 was resistant to penicillin and other *β-lactam* antibiotics and these agents degraded. Hence, *β-lactamase* might have been actived in PGB1. A phylogenetic comparison analysis revealed that PGB1 *β-lactamases* was closely associated with the class A *β-lactamases* of *A. faecalis* ZD02 (Fig. 5a). The large class A *β-lactamases* were divided into the subclasses A1a, A1b, A1c, and A2a [31]. Amino acid sequences of eight subclass A1b *β-lactamases* from *A. faecalis* strains (Fig. 5b) were compared and the results showed that PGB1 *β-lactamases* are categorized as class A, subclass A1b, and cluster LSBL3 mainly based on RTG located at positions 234–236..

## Discussion

Over the past ten years next-generation sequencing (NGS) platforms have dominated the genome sequencing market as they have high throughput and are cost-effective. In terms of de novo whole-genome assembly, however, NGS sequencing produces highly fragmented assemblies because it generates long repetitive regions [35, 36]. Long reads must be available when performing whole-genome assembly so that the repetitive elements are covered and potential assembly barriers are avoided [40, 41]. Here, the *A. faecalis* PGB1 genome was assembled de novo (4.45 Mbp) using ONT long reads combined with Illumina reads.

To define the taxonomic status of *A. faecalis PGB1*, we generated a core gene list based on the bacterial genome and plotted a phylogenetic tree based on that list. Modern microbial taxonomies are founded mainly on 16S rRNA gene relationships but have several limitations such as low phylogenetic resolution at the highest and lowest taxonomic ranks [41]. A core genome-based phylogenetic tree was successfully constructed [42] and accurate taxonomic definitions could be defined by the ModelFinder method. This procedure has certain advantages over 16S rRNA-based bacterial subtype taxonomies. As genome sequencing technology progresses, additional bacterial species will be routinely described based on their genome sequences. IQ-TREE is a reliable analytical software that could plays critical roles in taxonomic development and genomic analyses. It is known that phylogenic network [43, 44], instead of phylogenetic tree might present the tree evolutionary relationships of bacteria due to the frequent reticulate events like horizontal transfer. We will explore phylogenetic networks in the future.

We used the COG database to annotate the genome. COG is a popular tool for microbial genome annotation and functional classifications. It predicts alternative enzyme forms and assigns functional categories. Here, COG predicted 4,213 genes and assigned them to 21 functional categories. The defense mechanism-related genes were explored by identifying two cluster regions within the chromosome in the ranges of 2.5–2.6 M and 3.0–3.1 M (Fig. 2, Additional file 1 Tables S2).

Antibiotic resistance occurs in numerous bacterial species and poses a serious threat to human health. Therefore, we annotated antibiotic resistance genes in this study.We predicted antibiotic resistance genes using the CARD and AMR datasets. Both the chromosomes and the plasmids harbored antibiotic resistance genes. CARD and AMR identified the *aph(3'')-Ib* gene in plasmids and we established that it confers acquired antibiotic resistance. However, the predicted genes on the chromosomes did not confer acquired antibiotic resistance. AMR identified *aph (3')* and *bla*. It was already known that *A. faecalis* PGB1 is resistant to penicillin which is a β-lactam antibiotic. Therefore, we believe that AMR provided reliable results here.We compared our antibiotic resistance genes with ZD02 and found the resistance-nodulation-cell division (RND) antibiotic efflux pump gene *adeF* in both bacterial chromosomes. We identified 445 genes in the ZD02 chromosome and classified most of them in the antibiotic efflux, target alteration, and inactivation families. This configuration was similar for PGB1. However, four genes identified in ZD02 were not detected in PGB1.

There is evidence that > 50% of all predicted genes regulate the antibiotic efflux resistance mechanism [45]. We predicted that this mode of action applies to *A. faecalis PGB1*. There were 122 genes regulating the resistance-nodulation-cell division antibiotic efflux pump, 91 in the antibiotic efflux pump major facilitator superfamily (MFS), and 77 governing the ATP-binding cassette (ABC) antibiotic efflux pump. The RND superfamily comprises major multidrug-resistant efflux pumps and plays major roles in the acquisition and expression of the multidrug resistance phenotype [46]. The results of the present study suggest that the latter mechanism predominates in PGB1.

The 77 genes classified into the antibiotic target alteration mechanism group were further subdivided into 26 families. Of these, phosphoethanolamine transferase, glycopeptide resistance gene cluster, and antibiotic resistant isoleucyl-tRNA synthetase were the three largest families and they included 17, 7, and 6 genes, respectively. The newly sequenced PGB1 genome contained various genes with different multi-drug resistance functions enabling this bacterium to survive in highly concentrated antibiotic environments.

Bacteria acquire ARGs by insertion sequence [47] and phage [48] and by communication with their ambient environment. Our IS prediction revealed a similar Tn3-dependent IS pattern in various *A. faceliea* strains and a unique IS*3* pattern in PGB1. The two phages identified in the predicted ARGs were evidence for active communication between PGB1 and its ambient environment.

## Conclusion

In this study, a newly isolated multidrug-resistant *A. faecalis* strain PGB1 was identified and cultured in the presence of various antibiotics. This work demonstrated the antibiotic degradation characteristics of PGB1. Its genome was hybrid assembled and systematic analysis annotated the gene function and classification. Two acquired antibiotic resistance genes in the PGB1 plasmid were identified by this study. MEGs like IS and phage were predicted and compared between *A. faecalis* strains.

## Materials and methods

### Chemicals and media

Penicillin G potassium (PG) (99% purity) was obtained from the North China Pharmaceutical Factory (NCPF), Hebei, China. Its molecular weight and formula were 372.48 g/mol and C_16_H_17_KN_2_O_4_S, respectively. The mineral salt (culture) medium (MSM) comprised (g/L): NH_4_NO_3_ (2.0), K_2_HPO_4_ (0.5), KH_2_PO_4_ (0.5), MgSO_4_·7H_2_O (0.5), NaCl (0.2), CaCl_2_·2H_2_O (0.1), FeSO_4_·7H_2_O (0.01), and MnSO_4_ (0.01). All chemicals used were of analytical grade and were purchased from Sinopharm Chemical Reagent Co. Ltd. (Shanghai, China).

### Bacterial isolation and penicillin G tolerance

The bacterial strain PGB1 was isolated from a penicillin waste dreg supplied by NCPF and cultured with penicillin G as its sole carbon source to detect its penicillin G tolerance concentration. A single colony was selected from waste dreg dilutions medium (containing 300 mg/L penicillin G potassium) and cultured on beef protein medium at 30 °C in the dark until a pure culture was obtained. PGB1 was identified according to its morphology and the analysis of its 16S rDNA sequence.

The beef protein medium was sterilized at 121 °C for 15 min, cooled to 40–50 °C, and combined with 0 mg/L, 50 mg/L, 300 mg/L, 500 mg/L, or 1,000 mg/L PG, respectively. A 50-mL PGB1 suspension was shaken at 150 rpm at 37 °C and its absorbance was measured in a spectrophotometer (UV-2600A, Unico (Shanghai) Instrument Co., Ltd.) at 600 nm, and using the beef protein medium as blank control.

### β-lactam antibiotic degradation and standard curve

Antimicrobial drug sensitivity is usually determined by the disk diffusion method [49–51]. Here, *Staphylococcus aureus* ACCC 01,334 (penicillin G potassium), *Bacillus subtilis* (cefalexin and cefradine), and *Micrococcus luteus* (type II medium; cefuroxime sodium) were selected to determine the probability of toxicity and used in the *β-lactam* antibiotic degradation assays according to the USP XI standards in the United States Pharmacopeia (USP) [52]. The Kirby-Bauer agar disk diffusion method was used [53], with slight modifications. A volume of 0.2 mL diluted inoculum was spread on beef extract peptone agar. Sterile ready-made disks were imbibed with the PG dilutions and set on the inoculated plates. The plates were then incubated at 30 °C for 24 h and observed for inhibition zones. The latter were then measured to determine relative *β-lactam* antibiotic degradation. The samples extracted at time zero served as positive controls.

With reference to the USP, 0.015 g PG was accurately weighed into a 50-mL volumetric flask to prepare 300 mg/L penicillin mother liquor. Standard penicillin dilutions of 210 mg/L, 150 mg/L, 90 mg/L, 30 mg/L, 12 mg/L, 6 mg/L, and 0.6 mg/L were prepared by diluting the mother liquor and plotting a standard curve representing the relationship between the penicillin concentration and the inhibition zone diameter. Each sample was prepared in triplicate. The curve equation was.

1 $$\mathrm{y }(\mathrm{lnC})\hspace{0.17em}=\hspace{0.17em}0.217\mathrm{x}\hspace{0.17em}+\hspace{0.17em}0.112; {\mathrm{R}}^{2}\hspace{0.17em}=\hspace{0.17em}0.9970.$$

The standard curves for the other antibiotics were determined by the aforementioned method and found to be.

$$\begin{array}{l}\mathrm{cefalexin}:\mathrm{ lnC}\hspace{0.17em}=\hspace{0.17em}0.164\mathrm{x}\hspace{0.17em}+\hspace{0.17em}0.929, {\mathrm{R}}^{2}\hspace{0.17em}=\hspace{0.17em}0.994\\ \mathrm{cefradine}:\mathrm{ lnC}\hspace{0.17em}=\hspace{0.17em}0.229\mathrm{x}-0.016, {\mathrm{R}}^{2}\hspace{0.17em}=\hspace{0.17em}0.998\\ \mathrm{cefuroxime sodium}:\mathrm{ lnC}\hspace{0.17em}=\hspace{0.17em}0.167\mathrm{x}\hspace{0.17em}+\hspace{0.17em}2.372, {\mathrm{R}}^{2}\hspace{0.17em}=\hspace{0.17em}0.995\end{array}$$

where C is the antibiotic concentration (mg/L) and x is the inhibition zone diameter (mm).

### Library preparation and sequencing

Genomic DNA (gDNA) was extracted with a QIAamp DNA mini kit (Qiagen, Hilden, Germany) according to the manufacturer’s instructions to applied for genome sequencing. Sheared DNA was used to make gDNA libraries with a NEBNext® Ultra II™ DNA Library Prep kit for Illumina® (E7370L; New England Biolabs (NEB), Ipswich, MA, USA) and NEBNext® Multiplex Oligos for Illumina® (E6609L; New England Biolabs (NEB), Ipswich, MA, USA) according to the manufacturer’s instructions. Briefly, 200 ng gDNA was sheared in 50 µL for 340 s in a Covaris M220 (Covaris, Woburn, MA, USA) for further study.

To remove small DNA fragments and purify DNA for Nanopore library preparation, 1 × Agencourt AMPure XP beads (Beckman-Coulter, Brea, CA, USA) were added. The DNA library was prepared with an ONT 1D ligation sequencing kit (SQK-LSK108, ONT, Oxford, UK) and a native barcoding expansion kit (EXP-NBD103, ONT, Oxford, UK) according to the manufacturer’s protocols. Briefly, the gDNA was mechanically sheared in a g-TUBE (Covaris, Woburn, MA, USA) to an average 8 kb fragment length. The library sequencing procedure was conducted according to the manufacturer’s instructions.

### Genome assembly for Illumina and ONT datasets

After adapter trimming and parameter-based filtering, the Illumina sequence reads were assembled with Soapdenovo [54] with default parameters. Various k-mer sizes [31, 55, 56] were used to evaluate the assembly results and the best was selected and used in the subsequent analysis. The quality of each assembly was assessed based on N50 and total length.

The ONT long-read assemblies were constructed with Flye [57] using the default parameters. After the assembly, Pilon polished the results using the Illumina sequencing data and the ONT assembled scaffold reads [40]. CheckM v. 1.0.13 assessed assembly completeness and heterogeneity [58]. The marker gene set was evaluated with the program default dataset checkm_data_2015-01–16. The command [checkM lineage_c_Betaproteo bacteria (UID3959) fasta < bin folder >  < output folder >] was run to generate the result in one step. The input format was FASTA and the input bin folder contained a single genome and plasmid-like DNA sequence assembly file.

### Genome annotation and phylogenetic analysis

The genome sequence was predicted with the default setting in Prodigal and functionally analyzed online with eggNOG-mapper [59]. The mapping mode was DIAMOND, the taxonomic scope was automatically adjusted, and all orthologs and non-electronic terms were used. Subunit 5 s, 16 s, and 23 s ribosomal RNAs were extracted from the assembled *A. faecalis* PGB1 genome with RNAmmer v. 1.2 [60]. Non-coding RNA genes were predicted with Infernal [61]. The input file contained complete genomic and plasmid-like DNA sequences. Rfam was the RNA alignment database. A circular map displaying the genome and plasmid assemblies was plotted with Circos [62]. Defense mechanism-associated genes identified by eggNOG were indicated on the circular map.

A core genome phylogenetic tree was plotted with IQ-TREE [50, 63] which uses a model determined by ModelFinder. The genes of the selected strains were predicted with Prokka software and the core gene list was generated with Roary software. Only genes common to all genomes were used in the core genome analysis. The tree was plotted with 19 published reference genomes and a neighbor-joining (NJ) algorithm. *Escherichia coli* served as the outgroup (not shown). Phylogenetic trees were visualized with MEGA v. 6.0 [64].

### Antibiotic resistance gene analysis

The genes predicted by eggNOG were classified into various antibiotic types. The insertion sequences were analyzed with ISfinder (https://www-is.biotoul.fr/blast.php). The antibiotic resistance genes were predicted with the bacterial antimicrobial resistance reference gene database (https://www.ncbi.nlm.nih.gov/bioproject/PRJNA313047) and AMRFinderPlus (https://www.ncbi.nlm.nih.gov/pathogens/antimicrobial-resistance/AMRFinder/). Acquired antibiotic resistance genes were predicted with ResFinder and phages were identified by PHASTER. The *A. faecalis PGB1 β-lactamase* amino acid sequence and other *β-lactamase* nucleotide sequences in the NCBI database comprised classes A–D *β-lactamases *[31, 56] that were used to plot a phylogenic tree with MEGA v. 6.0 [64]. The phylogenetic tree was plotted by 1,000 bootstrap re-samplings and the maximum likelihood method. The *β-lactamase* sequences were aligned with ClustalX v. 2.1 [65]. The *β-lactamase* amino acid sequences within the same species and within a reference were compared to identify conserved regions [31].

## Supplementary Information

**Additional file 1.**

**Additional file 2.**

## Data Availability

The raw data and the assembly results for this genome project were deposited in the NCBI sequence read archive database under accession number PRJNA532790.

## Abbreviations

ARG: Antibiotic resistance gene
BEM: Beef extract-peptone medium
BLAST: Basic local alignment search tool
COG: Clusters of orthologous groups
EC: European Commission
gDNA: Genomic DNA
GO: Gene ontology
KEGG: Kyoto Encyclopedia of Genes and Genomes
LSBL: Limited-spectrum β-lactamases
NCBI: National Center for Biotechnology Information
NGS: Next-generation sequencing
ONT: Oxford Nanopore Technologies
PacBio: Pacific Biosciences
PCR: Polymerase chain reaction
RKN: Root-knot nematode
rRNA: Ribosomal RNA
TGS: Third-generation sequencing
tRNA: Transfer RNA
USB: Universal serial bus
