# Supplementary material for: Genomic and resistome analysis of Alcaligenes faecalis strain PGB1 by Nanopore MinION and Illumina Technologies
Source: BMC Genomics. 2022 Apr 20;23(Suppl 1):316. doi: 10.1186/s12864-022-08507-7 (PMC9022240; doi:10.1186/s12864-022-08507-7)
Supplement: Supplementary file 1 — Additional file 1. [file 12864_2022_8507_MOESM1_ESM.pdf]

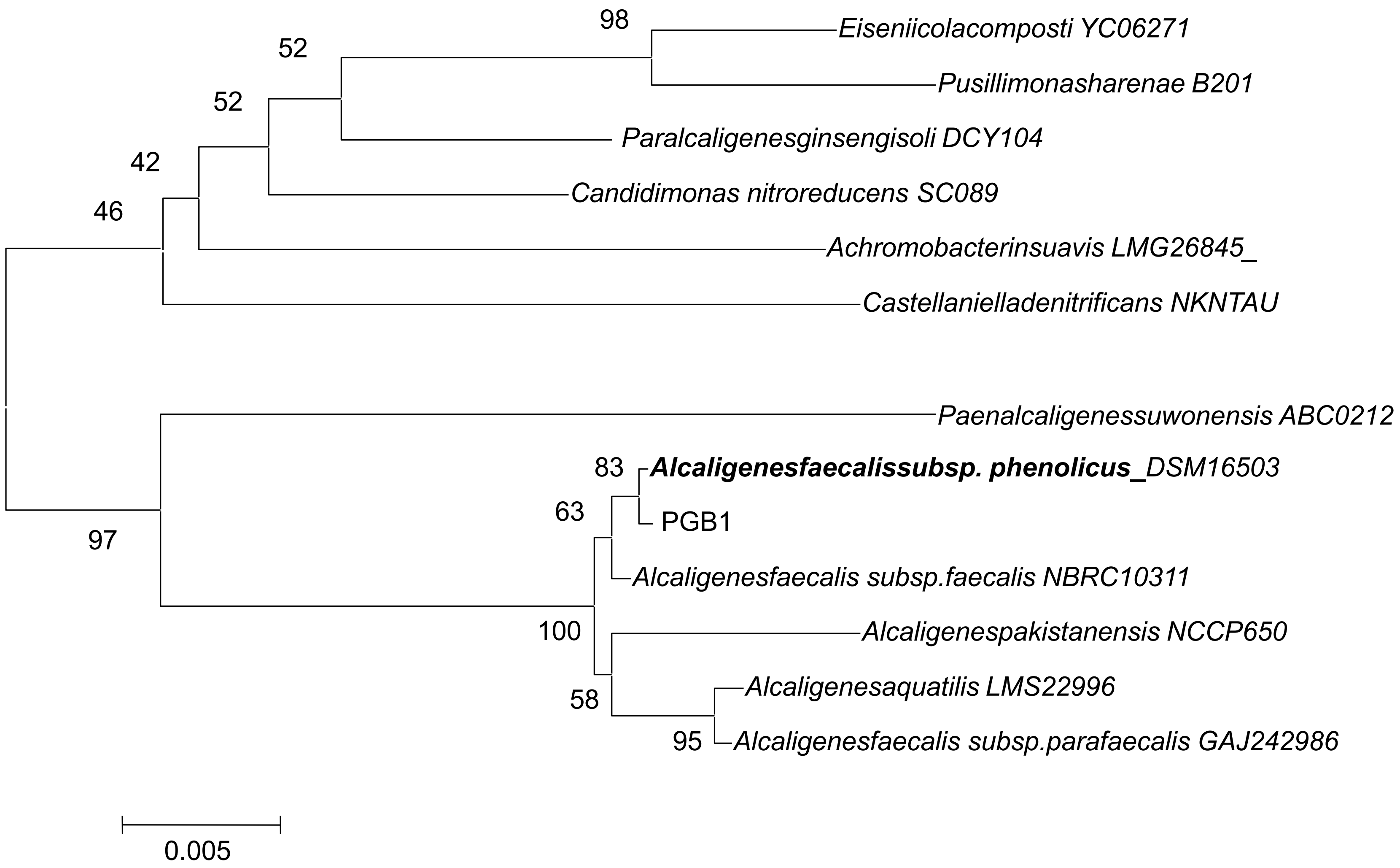

Figure S1. The phylogenetic tree of strain PGB1 based on 16S rDNA sequence.

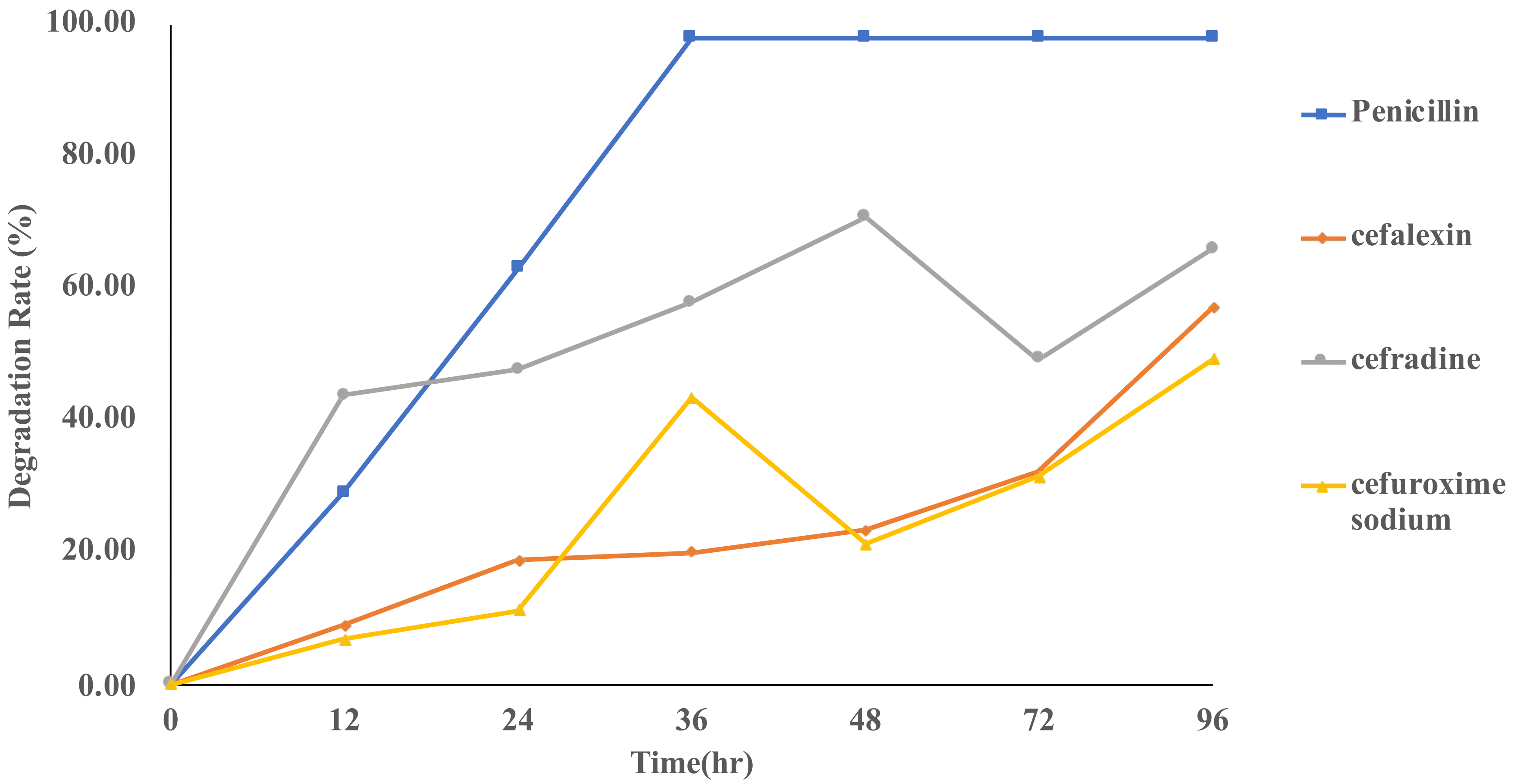

Figure S2. The degradation rate of *Alcaligenes faecalis* Strain PGB1 under four different beta-lactam antibiotics at concentration of 50mg/L.
